# Frontiers of marine science

**DOI:** 10.1098/rsbl.2010.1120

**Published:** 2011-01-05

**Authors:** Thomas J. Webb, Elvira S. Poloczanska

**Affiliations:** 1 Department of Animal and Plant Sciences, University of Sheffield, Sheffield S10 2TN, UK; 2 Climate Adaptation Flagship, CSIRO Marine and Atmospheric Research, EcoSciences Precinct, GPO Box 2583, Brisbane, Queensland 4001, Australia

**Keywords:** ecosystem model, climate, retrospective data, collaboration

## Abstract

On 9–13 October 2010 early career scientists from the UK and Australia across marine research fields were given the opportunity to come together in Perth, Australia to discuss the frontiers of marine research and exchange ideas.

## 1. Introduction

Many of the challenges that face twenty-first century scientists, such as climate change and ecosystem research, are inherently interdisciplinary in nature [1]. Perhaps nowhere is this better illustrated than in marine science, where the physics and chemistry of the medium are inextricably linked with the biology and ecology of ecosystems. Numerous feedback loops exist within and between biology and marine and atmospheric climate, which we are only beginning to understand, e.g. [2,3]. In addition, our marine environment is under considerable stress, with every square kilometre of the global ocean affected by anthropogenic drivers of ecological change [4]. Climate change is fundamentally altering marine systems, bringing challenges and costs for human societies and placing urgency on the science community to provide the information and understanding to drive policy and management responses [5]. Synergistic effects between climate and other anthropogenic stressors such as pollution and exploitation are likely to exacerbate climate change impacts in the oceans [6,7]. Marine systems also face the unique threat of ocean acidification as atmospheric CO_2_ levels increase [8].

The UK–Australia Frontiers of Science conference was held in October 2010 in Perth, Western Australia, supported by the UK's Royal Society and the Australian Academy of Science. The meeting brought together 70 early career scientists (35 from each country) over 3 days to present the latest advances in their fields, learn about research at the cutting edge of other disciplines, and explore new opportunities for international and multidisciplinary collaboration. Australia and the UK have an extensive and interlinked history and both countries are considered maritime nations with their oceans contributing substantial social and economic wealth [9,10]. It is therefore appropriate that these two countries came together to consider the interconnectedness of the world's marine ecosystems, and the interdependence of methods used to study and manage these environments.

## 2. Pathways to understanding the marine environment

**Figure 1.**
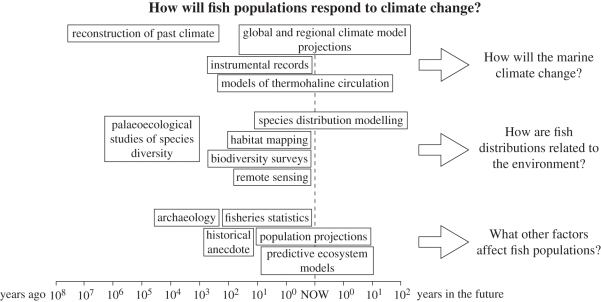
Questions in marine science are inherently interdisciplinary. For example, to address the question, *How will fish populations respond to climate change?* requires an understanding of climate past, present and future, and how environmental factors such as ocean chemistry, productivity and physical forcing influence the current distributions of fish and their predators and prey. In addition, understanding of other pressures on fish populations will be essential. These may include past and present exploitation in fisheries as well as probable responses to a range of policy scenarios, and may involve the complementary expertise of archaeologists, historians, social and political scientists. Each of these different disciplines will bring its own methods, including both empirical and modelling approaches. This interdisciplinary approach covers the requisite range of scales in space (from individual fish populations to global climate) and time (from deep time to the near future).

Frontiers of Science meetings are structured around a series of discipline-themed sessions, with three presentations setting out the state of the art in a given subject, and a strong emphasis on discussion among the multi-disciplinary audience. Each member of the organizing committee proposed important topics relevant to their theme for wider consideration and one topic was then selected for each disciplinary session. Even at the planning stage of this meeting, however, the inherent interdisciplinarity of marine science was evident. For example, ocean acidification was formally presented in the macrobiology session, but could equally have been placed in any of a number of different sessions, from climatology to chemistry to applied ecology. This problem-centred approach to science typically is indifferent to traditional disciplinary boundaries (figure 1), and in addition blurs the distinction between ‘pure’ and ‘applied’ research.

As well as the interdisciplinary nature of topics such as ocean acidification, ocean circulation and geoengineering, a number of other common themes linked the diverse sessions in the meeting. In particular, the interdependence of physical, chemical and biological processes across spatial and temporal scales as well as the consequent complementarity of methodological approaches applied by researchers.

### (a) Space

The title of the mathematics session, *Small things matter*, referred specifically to the role of eddy-scale turbulence within physical oceanography, and in particular to the importance of considering such small-scale processes in regional and global climate models. But the same sentiment summed up the microbiology session on *Symbioses*, which highlighted the vital, and often poorly understood, role that microbes play in ocean ecosystems, in part through their intricate relationships with multicellular organisms. Likewise, the Geotraces programme (www.geotraces.org), introduced in the chemistry session, seeks to understand the distribution of minute concentrations of the trace metals which underpin global biogeochemical cycles. Of course, macro-scale processes also exert a powerful influence on local phenomena (e.g. low frequency climate signals) and studies at this scale provide unique but necessary understanding in the context of global change [11].

### (b) Time

The meeting involved delegates with primary interests in documenting the past, understanding the present and predicting the future of the marine environment. The interdependence of these three perspectives was abundantly clear. For instance, information from the past can be used to inform our predictions of the future and develop hypotheses for testing in models and experiments. Retrospective data, for example from sediments and coral cores, can provide evidence of past climate or ecosystem states. However, palaeo-ecologists also require information from analogous extant species to inform their understanding of the fossil and sub-fossil record. One of the challenges highlighted at the meeting is the need to extend the temporal and spatial coverage of retrospective data. Such data are required by climate system models, in order to enhance our understanding of climate system dynamics, and by ecosystem models which aim to predict climate impacts. Whole ecosystem models can also be used to simulate conditions in the past or produce predictions of the future. Such models can simulate the state of ecosystems without exploitation or other anthropogenic pressures, conditions often beyond our data records, so expanding our understanding of key processes [12].

### (c) Methods

A major message from this meeting was the interdependence of theoretical and empirical approaches. Models have been developed across a variety of scales to give a global picture (e.g. physical and biological oceanography, modelling ocean- or global-scale circulation) or more complex local detail (e.g. eddy-scale processes, ecosystem dynamics). But the importance of empirical studies remains key, for verifying model predictions, for deriving parameter estimates and for suggesting future theoretical developments. Coordinated large-scale empirical programmes in the marine environment have been designed to improve the spatial coverage of our understanding of patterns in the biodiversity (e.g. the Census of Marine Life, www.coml.org) and chemical composition of the oceans (e.g. Geotraces, www.geotraces.org) as well as to document the history of the Earth system (e.g. the Integrated Ocean Drilling Program, www.iodp.org). Each of these multinational, multidisciplinary initiatives blurs the boundaries between theory and empiricism, with models driving empirical questions, and the resultant data feeding back into improved models of marine systems. A strength of the Frontiers of Science was to bring together modellers and empiricists in discussions focused on generic problems, rather than on specific methodologies. This approach offers the best pathway to understanding the marine environment.

## 3. Pushing the frontiers

Marine scientists face the challenges of working in a medium that can be difficult to access and sample, with large areas of the ocean still almost untouched by scientific surveys, e.g. [13,14]. Nonetheless, this conference highlighted how collaborations and technological advances are pushing the frontiers of marine science. Cutting-edge technologies are allowing us to collect information over large areas (e.g. ocean colour by satellite remote-sensing), thus allowing automated observing of marine life. Metagenomics, which link ‘old’ single-species empirical technologies and ‘new’ molecular biologies at community and ecosystem levels, have the potential to integrate across diverse fields that may have previously lacked a genetics perspective. The development of the kinds of multinational, interdisciplinary networks of marine researchers described above is rapidly advancing our understanding of the exchanges between oceanic physical and biological processes. Finally, the way we manage the marine environment is changing moving from single-species management to whole-ecosystem management, and ecosystem models which have the capacity to link physics, biology and societal goals can provide unique insight for managers [15].

## 4. To conclude

Our oceans cover 70 per cent of the Earth's surface and provide a suite of ecosystem services that are essential to human societies, economies and well-being but are increasingly under threat. Understanding marine systems, and in particular predicting how they will respond to environmental change, demands cooperation across disciplines. Marine scientists, with already shared vocabulary, and some history of collaboration (e.g. shared cruises), are well placed to pioneer partnerships across the natural and physical sciences. The Frontiers of Marine Science meeting not only encouraged the participants to think more broadly across disciplines, but hopefully fostered new collaborations and new thinking both within and between the two countries and across disciplines.
